# Neurite-Localized mRNA Transport Genetics Shapes Clinical and Brain Features in Schizophrenia

**DOI:** 10.1192/j.eurpsy.2026.10737

**Published:** 2026-07-31

**Authors:** C. Almodóvar-Payá, D. Herrera-Escartín, K. Roy, M. Latorre-Guardia, N. Hostalet, B. García-Ruíz, M. Guardiola-Ripoll, P. Fuentes-Claramonte, B. Chaumette, M. Moreira, M. Giralt-López, S. Sarró, R. Salvador, E. Pomarol-Clotet, M. Aldea, C. Gallego, M. Fatjó-Vilas

**Affiliations:** 1 FIDMAG Germanes Hospitalàries Research Foundation; 2 Biomedical Research Networking Centre for Mental Health (CIBERSAM-G15); 3 Departament Biologia Evolutiva, Ecologia i Ciències Ambientals, Facultat de Biologia, Universitat de Barcelona; 4 FIDMAG Germanes Hospitalàrias Research Foundation; 5 Institut Biologia Molecular de Barcelona -CSIC, Barcelona; 6 Institut d’Investigació Sanitària Pere Virgili (IISPV-CERCA); 7 Hospital Universitari Institut Pere Mata (HUIPM), Reus, Spain; 8 Institute of Psychiatry and Neuroscience of Paris (INSERM U1266), GHU-Paris Psychiatrie et Neurosciences, Université Paris Cité, Paris, France; 9 Servei de Psiquiatria Infantil i de l’Adolescència, Hospital Universitari Germans Trias i Pujol, Badalona; 10 Departament de Psiquiatria i Medicina Legal, Universitat Autònoma de Barcelona, Barcelona, Spain

## Abstract

**Introduction:**

Advances in psychiatric genetics link synaptic plasticity to schizophrenia (SZ), though specific mechanisms remain unclear. This process depends on rapid molecular regulation, where mRNA transport from soma to synapses via neurites plays a key role. Studying mRNAs localized in neurites may provide new insights into the involvement of mRNA transport and availability regulation processes in SZ.

**Objectives:**

We aimed: i) to calculate the SZ polygenic risk score based on neurite-localized transcripts and compare it between healthy controls and SZ patients; and, ii) to test its association with clinical variables and brain structure.

**Methods:**

We selected 70 neurite-localized transcripts, consistently identified in more than 15 transcriptomic studies comparing soma and neurite content (von Kügelgen et al., 2020). In a sample of 115 controls and 169 patients with SZ, we calculated the polygenic risk score (PRS) using PRSet, grouping SNPs near these transcripts (±10 kb). The resulting PRS (nPRS) was evaluated in relation to diagnosis, clinical variables (age at onset and PANSS scores), and brain structural measures (cortical surface area and thickness).

**Results:**

The nPRS did not differ between patients and controls, but it was associated with age at onset (β=5158.09, SE=2518.78, p=0.04) and showed a trend with the positive PANSS dimension scores (β=–4110.00, SE=2303.00, p=0.08). Additionally, an association was also observed between the nPRS and the surface area of the left frontal pole (β=39,800; SE=9,373; t=4.243; p<0.001) in patients.

**Conclusions:**

Our data suggest that the genetic load associated with neurite-localized mRNAs is related to clinical and brain features of SZ, supporting the hypothesis that transcript transport and regulation mechanisms play a relevant role in its pathophysiology.

**Acknowledgements:**

Instituto de Salud Carlos III through the PFIS contract (NH, FI21/00093), the Miguel Servet contract (MF-V, CP20/00072), JdC post-doctoral fellowship (MG-R, JDC2023-050677-I), Fundació Marató C/40/2022, grant funded by MICIU/AEI/10.13039/501100011033 and “ESF Investing in your future” (DH-E, PRE2021-100310)

**Disclosure of Interest:**

None Declared

